# Manic episode after treatment with ventriculoperitoneal shunting due to obstructive hydrocephalus with corpus callosal signal changes: a case report

**DOI:** 10.1186/1743-8454-7-S1-S50

**Published:** 2010-12-15

**Authors:** Liberto Brage-Martin, Agustin Castañeyra-Perdomo, Juan C Ribas-Neijerk, Mario García-Conde, Pablo Febles-Garcia, Mario Spreafico-Guerrero, Luis Pérez-Orribo, Rebeca Pérez-Alfayate, Emilia M Carmona-Calero, Victor García-Marín

**Affiliations:** 1 Servicio de Neurocirugía, Hospital Universitario de Canarias. La Laguna, Tenerife, Spain; 2 Departamento de Anatomía, Facultad de Medicina. La Laguna, Tenerife, Spain

## Background

The radiological changes that appear in the corpus callosum in patients with obstructive hydrocephalus, after treatment with a ventricle-peritoneal drainage, are a quite frequent finding in relation with the different series published with no clinical significance. The signal alteration showed by the magnetic resonance (MR) in this brain structure is associated with psychiatric diseases (bipolar disorder, schizophrenia, etc) without knowledge of physiopathological details. We present a case report of a patient with obstructive hydrocephalus who develops important psychiatric symptoms after being treated with a ventricle-peritoneal valve, with the radiological changes described above.

## Materials and methods

We present a 76 year old male patient who has had the Hakim-Adams triad for 7 months at the moment of consulting. Severe triventricular hydrocephalus was found by brain MR related to stenosis of the sylvian aqueduct. A ventriculoperitoneal shunt was implanted.

## Results

Clinical symptoms greatly improved after treatment with a ventricle-peritoneal system of medium pressure. A clinical control was performed by interviewing and doing CT scans, severe hypodensity was found in the genu of the corpus callosum. One year four months later, the patient developed a manic episode which consisted of irritability, restlessness, grandiose behaviour, decreased need for sleep and psychotic experiences such as hallucinations and paranoia. The brain MR performed at the time showed considerable hyperintensity in the genu of the corpus callosum.

## Conclusions

We suggest that if a patient develops psychiatric symptoms after undergoing a ventricle-peritoneal shunt, a brain MR must be done to look for signal changes in the corpus callosum, and if necessary recalibrate the pressure of the valve.

